# Antibacterial Activity and Safety of Oregano Oil–Lauric Acid Cationic Nanostructured Lipid Carriers in Nile Tilapia (*Oreochromis niloticus*)

**DOI:** 10.3390/ani16111639

**Published:** 2026-05-27

**Authors:** Ratima Kumanbut, Manoj Tukaram Kamble, Wanna Sirimanapong, Sirikorn Kitiyodom, Jakarwan Yostawonkul, Kim D. Thompson, Nopadon Pirarat

**Affiliations:** 1 M.Sc. Program in Research for Enterprise, Faculty of Pharmaceutical Sciences, Chulalongkorn University, Bangkok 10330, Thailand; ratimakuman@gmail.com; 2 Center of Excellence in Wildlife, Exotic, and Aquatic Animal Pathology, Faculty of Veterinary Science, Chulalongkorn University, Bangkok 10330, Thailand; maav.manya@gmail.com (M.T.K.); taregust@hotmail.com (S.K.); jond4kim@gmail.com (K.D.T.); 3 Veterinary Aquatic Animal Research & Health Care Unit, Department of Clinical Science and Public Health, Faculty of Veterinary Science, Mahidol University, Salaya Campus, Nakhon Pathom 73170, Thailand; wanna.sir@mahidol.ac.th; 4 National Nanotechnology Center (NANOTEC), National Science and Technology Development Agency (NSTDA), Pathum Thani 12120, Thailand; jo_jak49@hotmail.com

**Keywords:** Nile tilapia, oregano oil, lauric acid, nanostructured lipid carriers, *Streptococcus agalactiae*, antibacterial activity, acute toxicity, sustainable aquaculture

## Abstract

Aquaculture faces increasing challenges from bacterial diseases and the widespread use of antibiotics, which contribute to antimicrobial resistance and environmental concerns. Oregano oil–lauric acid cationic nanostructured lipid carriers (OE-L^+^NLCs) were developed in this study and their antibacterial activity and safety were evaluated in Nile tilapia (*Oreochromis niloticus*). The nanoparticles were prepared using a hot homogenization–ultrasonication method and characterized based on their particle size, surface charge, morphology, and release profile. Antibacterial activity was assessed against six isolates of *Streptococcus agalactiae* using minimum inhibitory and bactericidal concentration assays, while their stability was evaluated under different storage conditions and simulated gastrointestinal environments. In vivo safety was determined through median lethal concentration analysis, survival assessment, and histopathology. The developed OE-L^+^NLCs showed a mean particle size of 175.90 ± 4.94 nm, positive zeta potential of +44.02 ± 0.54 mV, and low polydispersity index (0.15 ± 0.00). The formulation exhibited sustained carvacrol release (70.46 ± 0.96% over 24 h) and strong antibacterial activity against all six isolates, with MIC and MBC values of 0.25 and 0.25–0.49 mg/mL, respectively. The nanoformulation maintained antibacterial activity during 90 days of storage and under simulated gastric conditions. In vivo toxicity assessment showed that OE-L^+^NLCs exhibited a higher LC_50_ value (11.11 mg/mL) than free oregano oil (5.89 mg/mL), together with improved survival and reduced histopathological alterations in Nile tilapia. Overall, the formulation provides an effective and stable delivery platform for natural antimicrobials, offering a promising alternative to antibiotics for sustainable disease management in aquaculture.

## Simple Summary

Fish farming is an important source of food worldwide, but disease outbreaks often lead to heavy production losses. Farmers commonly use antibiotics to control disease outbreaks, but these can cause serious problems such as drug resistance, environmental pollution, and risks to human health. Therefore, safer and more sustainable alternatives are needed. In this study, we developed a new delivery system that combines oregano oil, a natural plant extract with antibacterial properties, and lauric acid, a fatty acid known to kill harmful bacteria. These compounds were enclosed in tiny lipid particles to improve their stability and effectiveness. This formulation was highly effective against a major fish pathogen that causes disease in tilapia. The particles remained stable during storage and worked well under stomach-like conditions, although their effectiveness decreased under intestinal conditions. Safety tests in fish showed that the encapsulated formulation was less toxic than the non-encapsulated compounds. Overall, this study demonstrates that combining natural compounds with advanced delivery systems can provide a promising alternative to antibiotics in aquaculture, helping to improve fish health while reducing environmental and public health risks.

## 1. Introduction

Nile tilapia (*Oreochromis niloticus*) is among the most widely farmed freshwater fish species globally, with production reaching 5.3 million tonnes in 2022, owing to its rapid growth, omnivorous feeding habits, and tolerance to a wide range of environmental conditions [1,2,3]. Across tropical and subtropical regions, tilapia aquaculture contributes to food security, affordable protein supply, and the economic stability of rural communities [4].

However, as aquaculture systems intensify, the frequency and severity of infectious diseases have also increased. Among these, *Streptococcus agalactiae* has emerged as one of the most destructive bacterial pathogens in tilapia culture worldwide [5,6,7,8]. This Gram-positive, β-hemolytic bacterium causes streptococcosis, a systemic infection characterized by septicemia, exophthalmia, erratic swimming, and high mortality, sometimes exceeding 50% during severe outbreaks [9,10,11]. Such losses impose major economic and social burdens on small-scale and commercial farmers alike.

Antibiotics and chemotherapeutic agents remain the most common control measures against *S. agalactiae* [12]. While effective in the short term, their repeated and indiscriminate use has led to the emergence of antimicrobial-resistant strains [13,14], residue accumulation in fish tissues [15,16], and environmental contamination [17,18]. These consequences raise concerns about fish safety, human health, and the long-term sustainability of intensive aquaculture systems.

Vaccination provides a promising alternative, and several commercial vaccines have shown protective effects against *S. agalactiae*. Nevertheless, vaccine efficacy often varies depending on strain diversity, fish age, environmental stress, and the need for booster doses [19,20]. Moreover, vaccines may not offer rapid protection during acute outbreaks, underscoring the need for reliable, sustainable, and cost-effective antimicrobial strategies for tilapia health management [21,22].

Plant-derived essential oils have increasingly attracted attention as environmentally friendly antimicrobial agents in aquaculture [23,24]. Oregano (*Origanum vulgare* L.) essential oil (OE), rich in carvacrol and thymol, exhibits broad-spectrum antibacterial, antioxidant, and immunostimulatory activities against both Gram-positive and Gram-negative bacteria, including *Streptococcus* spp. [25,26,27,28]. However, its high volatility, low aqueous solubility, and susceptibility to oxidation significantly limit its stability and bioavailability in aquatic environments [29,30,31].

Lauric acid, a medium-chain fatty acid with potent antibacterial activity, particularly against Gram-positive pathogens, disrupts bacterial cell membranes through lipid bilayer destabilization and interference with membrane-associated functions [32,33]. Importantly, lauric acid can confer a cationic surface charge when incorporated into lipid-based nanocarriers, enhancing electrostatic interaction with negatively charged bacterial membranes and improving antimicrobial performance [34,35]. When co-formulated with essential oils, lauric acid may also facilitate synergistic membrane permeabilization, allowing lower effective doses of volatile compounds while mitigating cytotoxicity through controlled release [36,37].

Nanotechnology offers a promising approach to address these challenges by improving the encapsulation, protection, and controlled release of lipophilic compounds [38]. Nanostructured lipid carriers (NLCs), which consist of solid and liquid lipids stabilized by surfactants, can protect bioactive compounds from oxidation, enhance solubility, and enable sustained release over time [39]. In particular, cationic NLCs show stronger adhesion to negatively charged bacterial membranes and mucosal surfaces, which can improve antimicrobial activity and influence host–pathogen interactions [8,40,41,42].

However, despite the well-documented antimicrobial properties of oregano oil, lauric acid, and lipid-based nanocarrier systems individually, studies investigating their combined incorporation within cationic nanostructured lipid carriers for aquaculture applications remain limited. Previous studies have mainly focused on free oregano oil supplementation, nanoemulsions, or non-cationic delivery systems, which may exhibit limited stability, rapid release, or reduced interaction with negatively charged bacterial membranes and mucosal surfaces [43,44,45,46]. In addition, the synergistic antibacterial and safety effects of combining oregano oil and lauric acid within a cationic lipid nanocarrier against *S. agalactiae* in Nile tilapia have not been comprehensively evaluated. Therefore, we hypothesized that this cationic nanoformulation could enhance antibacterial activity, improve physicochemical stability, prolong the release of bioactive compounds, and reduce acute toxicity compared with non-encapsulated and non-cationic formulations.

Building on these advantages, the present study aimed to develop and characterize OE-L^+^NLCs and to evaluate their in vitro antibacterial performance against *S. agalactiae*. The study systematically assessed the physicochemical properties, carvacrol release behavior, and antibacterial activity of the formulations, together with their stability under different storage temperatures and simulated gastrointestinal digestion conditions. In addition, in vivo toxicity and safety were evaluated through LC_50_ determination, survival analysis (Kaplan–Meier and Cox regression), and histopathological examination of major organs in Nile tilapia. Collectively, this study presents an integrated approach for the stabilization and delivery of plant-derived antimicrobials using cationic nanocarrier systems, highlighting their potential as feed-compatible alternatives to antibiotics for sustainable aquaculture.

## 2. Materials and Methods

### 2.1. Experimental Design

This study was conducted in sequential phases to evaluate the development, antibacterial efficacy, stability, and safety of OE-L^+^NLCs. First, the nanoparticles were prepared and characterized for their physicochemical properties, release behavior, and in vitro antibacterial activity against *S. agalactiae*. Next, their stability and retained antibacterial activity were assessed under different storage temperatures and simulated gastrointestinal conditions. Finally, in vivo safety was evaluated in Nile tilapia through acute toxicity, survival analysis, and histopathological examination.

### 2.2. Chemicals

Oregano oil (OE), derived from *Origanum vulgare* L. (CAS No. 84012-24-8) and containing approximately 85% carvacrol and 3% thymol, was obtained from Katyani Exports (Delhi, India). Lauric acid cationic (L^+^; CAS No. 60372-77-2), originally patented in Spain [47] and now produced by Vedeqsa Lamirsa Group (Barcelona, Spain), was used as the cationic lipid. Medium-chain triglycerides (MCT) were purchased from Tulip Chemplast Co., Ltd. (Samut Sakhon, Thailand). Sorbitol monooleate (Span 20) and polysorbate 80 (Tween 80) were obtained from Merck Pte. Ltd. (Singapore). Montanov 82, polyethylene glycol, and glycerol monohydrate were sourced from Waruto Sama Co., Ltd. (Bangkok, Thailand). All reagents were of analytical grade and used without further purification.

### 2.3. Preparation of Oregano Oil–Lauric Acid Cationic Nanostructured Lipid Carriers

Nanostructured lipid carriers were prepared using a modified hot homogenization–ultrasonication method (Figure S1). Briefly, the oil phase consisted of OE (10 g) and MCT (10–15 g) mixed with surfactants (span 20 and montanov 82, 1–3 g each) and maintained at 50–60 °C with gentle stirring (150 rpm). The aqueous phase consisted of purified water, Tween 80, glycerol monohydrate, polyethylene glycol (each 1–3 g), and L^+^ (2 g), preheated to the same temperature. The aqueous phase was slowly added to the oil phase with continuous stirring, homogenized, and subsequently sonicated for 5 min using a Qsonica sonicator (35-amp pulse, 10 s on/5 s off cycles) following Yostawonkul et al. [48]. The resulting emulsion was cooled to room temperature to obtain the final OE-L^+^NLCs formulation. Control formulations included: (i) OE-NLCs (without L^+^ in the aqueous phase), (ii) L^+^NLCs (without OE in the oil phase), and (iii) blank NLCs (without both OE and L^+^).

For comparative purposes, an OE solution (dissolved in 0.5% DMSO) and an L^+^ solution (dissolved in sterile distilled water) were prepared. The composition of all formulations and solutions is summarized in Table S1. The prepared formulations were subsequently subjected to physicochemical characterization, release profiling, and antibacterial evaluation, as described in the following sections.

### 2.4. Bacterial Strains and Culture Conditions

Six *S. agalactiae* strains, PC08 (Ia), SM81 (Ia), CT59 (III), SM86 (III), SM24 (Ia), and AY19 (Ia), were isolated from the brain tissue of diseased Nile tilapia collected from farms in Phetchaburi, Samut Sakhon, Chanthaburi, and Ayutthaya provinces, Thailand. Isolates were subcultured on Brain Heart Infusion (BHI) agar (pH 7.2) and incubated at 30 °C for 24 h. Bacterial suspensions were prepared in BHI broth under shaking conditions (150 rpm, 24 h). Cell density was standardized spectrophotometrically (OD_600_ = 0.2), corresponding to approximately 1 × 10^8^ CFU/mL. Culture purity was confirmed by Gram staining and microscopic examination prior to use.

### 2.5. Physicochemical Characterization of Nanoparticles

The physical appearance (color, homogeneity, and phase separation) of all formulations was visually assessed after 24 h of equilibration at room temperature. Particle size, zeta potential, and polydispersity index (PDI) were measured using dynamic light scattering (Zetasizer Ultra, Malvern Panalytical Ltd., Malvern, UK) at 25 °C. Measurements were performed in triplicate.

Nanoparticle morphology was examined using a transmission electron microscope (TEM; Talos F 200X, Thermo Fisher Scientific, Waltham, MA, USA). Samples were diluted (1:50, *v*/*v*) with deionized (DI) water, stained with 1% uranyl acetate, air-dried on carbon-coated grids, and imaged at an accelerating voltage of 200 kV [49].

### 2.6. Encapsulation Efficiency, Drug Loading and In Vitro Release of Oregano Oil

The encapsulation efficiency (EE) and drug loading (DL) of carvacrol, the major bioactive compound of oregano oil, in OE-L^+^NLCs and OE-NLCs were determined using an ultrafiltration–centrifugation method. Briefly, nanoparticle suspensions were transferred into Amicon Ultra-15 centrifugal filter units (Merck Millipore Ltd., Darmstadt, Germany) and centrifuged at 8000× *g* for 1 h to separate free carvacrol from encapsulated fractions. The filtrate containing non-encapsulated carvacrol was passed through a 0.22 μm nylon membrane filter and quantified using gas chromatography coupled with flame ionization detection (GC-FID; Agilent Technologies 7890A, Agilent Technologies Inc., Santa Clara, CA, USA) following previously reported methods [48,50]. Encapsulation efficiency and drug loading were calculated according to the following equations:

% EE = [(C_i_ − C_f_)/C_i_] × 100 (1)

% DL = [(W_i_ − W_f_)/W_NLC_ × 100 (2)

where C_i_ represents the initial concentration of carvacrol added to the formulation, C_f_ represents the concentration of non-encapsulated carvacrol in the filtrate, W_i_ represents the initial weight of carvacrol added, W_f_ represents the weight of non-encapsulated carvacrol, and W_NLC_ represents the total weight of the nanostructured lipid carrier formulation.

The in vitro release profile of carvacrol from OE-L^+^NLCs, OE-NLCs and OE solution was evaluated using the dialysis bag diffusion method described by Yostawonkul et al. [51]. Aliquots containing an amount equivalent to 10% (*w*/*v*) of carvacrol solubility were placed in dialysis membranes (MWCO 10 kDa) and immersed in phosphate-buffered saline (PBS, pH 7.4) supplemented with 0.5% Tween 80. Samples were incubated at 37 °C with constant stirring (40 rpm). At predetermined time points (0.2, 0.5, 1, 2, 6, 12 and 24 h), aliquots were withdrawn, filtered (0.22 µm), and analyzed for carvacrol content using gas chromatography–flame ionization (GC-FID) according to Lee et al. [52]. The cumulative release (%) was calculated as:

Cumulative release (%) = 100 − (% carvacrol remaining)(3)

### 2.7. Antibacterial Activity Assays

Antibacterial activity was evaluated under three conditions: baseline, post-storage, and post-digestion.

#### 2.7.1. Baseline Antibacterial Activity Assays

Baseline antibacterial activity of OE-L^+^NLCs, OE-NLCs, L^+^NLCs, blank NLCs, OE solution, and L^+^ solution was evaluated against six *S. agalactiae* isolates using the broth microdilution method in accordance with NCCLS guidelines [53,54]. Stock solutions (125 mg/mL) were serially two-fold diluted in Muller–Hinton broth (MHB) to obtain concentrations ranging from 62.50 to 0.13 mg/mL. Each well of a 96-well microplate contained 100 µL of the diluted formulation and 100 µL of bacterial suspension adjusted to approximately 1 × 10^6^ CFU/mL, resulting in a final bacterial concentration of approximately 5.0 × 10^5^ CFU/mL/well, and a final concentration of sample solutions ranging from 31.25 to 0.06 mg/mL. Enrofloxacin (5 µg/mL) served as a positive control, while sterile water or 0.5% DMSO served as negative controls. Plates were incubated at 30 °C for 24 h. The minimum inhibitory concentration (MIC) was defined as the lowest concentration showing no visible growth. Minimum bactericidal concentration (MBC) was determined by subculturing aliquots from clear wells onto BHI agar and identifying the lowest concentration preventing colony formation after 24 h (Figure S2).

#### 2.7.2. Antibacterial Activity After Thermal Storage

To assess antibacterial activity following storage, the MIC and MBC values of all formulations were re-evaluated against *S. agalactiae* strain AY19 after storage at different temperatures and for varying durations, as described in Section 2.8. Strain AY19 was selected as a representative isolate based on its consistent growth characteristics and reproducible response in preliminary assays.

#### 2.7.3. Antibacterial Activity After Simulated Digestion

Antibacterial activity of digested formulations obtained after simulated gastric and intestinal phases (see Section 2.9) was evaluated against *S. agalactiae* strain AY19 using the same broth microdilution protocol as described.

### 2.8. Thermal Stability Study

OE-L^+^NLCs and control formulations were stored at 4 °C (refrigeration), 25 °C (room temperature), and 37 °C (physiological temperature) for 90 days [55]. To minimize contamination and environmental exposure during long-term storage, all formulations were prepared from a single batch and pre-aliquoted into sterile tubes according to each storage condition and sampling time point. Each aliquot was opened only once at the designated time point using aseptic conditions. Samples were analyzed at 0, 30, 60, and 90 days for physical appearance, particle size, zeta potential, and PDI. At each sampling interval, antibacterial activity against *S. agalactiae* strain AY19 was evaluated as described in Section 2.7.2 using freshly prepared culture media under standardized assay conditions. This analysis enabled evaluation of the relationship between physicochemical stability and retained antibacterial activity.

### 2.9. In Vitro Simulated Digestion

Nanoparticle stability under gastrointestinal conditions was evaluated using simulated gastric fluid (SGF) and simulated intestinal fluid (SIF). SGF (2 g/L NaCl, pH 2.0) and SIF (6.8 g/L KH_2_PO_4_, pH 7.0) were freshly prepared. Pepsin (10 mg/mL) and pancreatin (10 mg/mL) were added to SGF and SIF, respectively [56]. For the gastric phase, 10 mL of each formulation was mixed with 9 mL SGF and incubated at 37 °C and 100 rpm for 2 h. For the intestinal phase, 10 mL of the gastric digest was transferred into 9 mL SIF containing 160 mg bile salts and incubated for an additional 2 h under identical conditions (Figure S3). Following digestion, samples were centrifuged (1000× *g*) to remove insoluble material. The supernatant was analyzed for particle size, zeta potential, and PDI. Antibacterial activity against *S. agalactiae* strain AY19 was evaluated to assess bioactivity retention (Figure S3). This simulated digestion model was used to assess whether nanoparticle integrity and antibacterial efficacy were maintained under gastrointestinal conditions.

Although the digestion experiments were conducted at 37 °C, which is higher than the typical physiological temperature range of Nile tilapia (approximately 28–30 °C), this condition was selected to maintain consistency with standardized in vitro digestion protocols frequently used in nanoencapsulation and lipid-based nanocarrier studies. This approach facilitates comparison with previously published studies and ensures reproducibility of enzymatic digestion conditions.

### 2.10. Experimental Design for Acute Toxicity Assessment

Healthy juvenile monosex (male) Nile tilapia (mean weight 10.0 ± 2.0 g; mean length 8.6 ± 1.2 cm) were acclimated for 14 days in 250 L fiberglass tanks with continuous aeration. Water quality parameters were maintained at 28–30 °C, pH 7.0–7.5, dissolved oxygen ≥ 5 mg/L, with a daily 20% water exchange.

During acclimation, fish were fed a commercial pellet diet at 3% of body weight per day. Following acclimation, fish were randomly assigned to six formulation groups (OE-L^+^NLCs, OE-NLCs, L^+^NLCs, OE solution, L^+^ solution, and blank NLCs), with each group receiving nine concentrations of their assigned formulation (100.0, 50.0, 20.0, 10.0, 5.0, 3.3, 2.5, 2.0, and 0 mg/mL [control]). Each concentration was evaluated in triplicate, with 10 fish used per replicate (30 fish per concentration) [57]. This resulted in 270 fish per formulation and a total of 1620 fish used in the acute toxicity experiment.

Each formulation was mixed with feed at approximately 30% moisture, air-dried on trays, and coated with 1% squid oil to minimize leaching of active compounds during feeding. Prepared diets were stored at 4 °C and used within 7 days. Control groups received feed treated with distilled water and squid oil to account for potential coating effects.

Fish were hand-fed the experimental diets at 5% of body weight per day, given as three equal feedings. Initial body weight was recorded to adjust feeding rates. Fish were monitored for mortality and clinical signs over a 96 h exposure period [58]. Mortality was defined as the absence of opercular movement and no response to gentle stimulation. The LC_50_ (median lethal concentration) was calculated using Probit analysis based on cumulative mortality across treatments.

### 2.11. Histopathological Evaluation of Dietary Toxicity in Nile Tilapia

At 96 h post-feeding, tissue samples (*n* = 6 per group) were collected from the intestine, liver, kidney, spleen, brain, and gills of randomly selected fish. Prior to sampling, fish were anesthetized with clove oil (20 mg/L) [59]. Collected tissues were fixed in 10% neutral buffered formalin for 24 h, followed by dehydration through a graded ethanol series (70% to absolute ethanol). Samples were then cleared in xylene and embedded in paraffin wax. Tissue sections (4–5 µm thick) were prepared and stained with hematoxylin and eosin for histopathological evaluation, following the method described by Moustafa et al. [60].

### 2.12. Statistical Analysis

All data were expressed as mean ± standard deviation (*n* = 3) and analyzed using SPSS version 29 (IBM Corp., Armonk, NY, USA). Normality and homogeneity of variances were verified prior to analysis. Independent samples *t*-tests were used to compare EE and DL between OE-L^+^NLCs and OE-NLCs formulations. One-way ANOVA followed by Duncan’s multiple range test was used to compare physicochemical characteristics and antibacterial activity among formulations. Two-way ANOVA was used to assess the effects of storage temperature and duration on nanoparticle stability, while repeated-measures ANOVA evaluated differences in carvacrol release profiles over time. Acute toxicity was assessed using probit analysis to estimate median LC_50_ values together with slope parameters and confidence intervals, based on dose–response relationships. Survival data were analyzed using the Kaplan–Meier method to estimate survival probabilities over time, and differences among treatment groups were evaluated using the log-rank test. Additionally, Cox proportional hazards regression analysis was performed to determine the relative mortality risk among formulations and to quantify hazard ratios. A *p*-value < 0.05 was considered statistically significant.

## 3. Results

### 3.1. Physicochemical Characterization of Nanoparticles

All formulated preparations, including OE-L^+^NLCs, OE-NLCs, L^+^NLCs, and blank NLCs, remained physically stable at 25 °C after 24 h, showing no visible phase separation or aggregation. Both OE-L^+^NLCs (Figure S4A) and OE-NLCs (Figure S4B) appeared as opaque cream emulsions, while L^+^NLCs (Figure S4C) and blank NLCs (Figure S4D) were white and homogeneous. In contrast, the OE solution (Figure S4E) was brown and water-insoluble, whereas the L^+^ solution (Figure S4F) was transparent and fully soluble in water.

The mean particle size of the nanoparticle formulations ranged from 151.67 ± 3.51 nm to 175.90 ± 4.94 nm, all within the nanoscale range suitable for feed delivery applications (Table 1). In contrast, unencapsulated OE and L^+^ solutions exhibited larger particle sizes (>230 nm) and high heterogeneity (PDI > 0.3). The OE-L^+^NLCs and L^+^NLCs exhibited strongly cationic zeta potentials of +44.02 ± 0.54 mV and +42.12 ± 0.66 mV, respectively, indicating excellent colloidal stability and potential interaction with negatively charged bacterial membranes and fish mucosal surfaces. Conversely, OE-NLCs and blank NLCs showed anionic charges (−41.69 ± 1.62 mV and −35.95 ± 0.32 mV, respectively).

**Figure 1:**
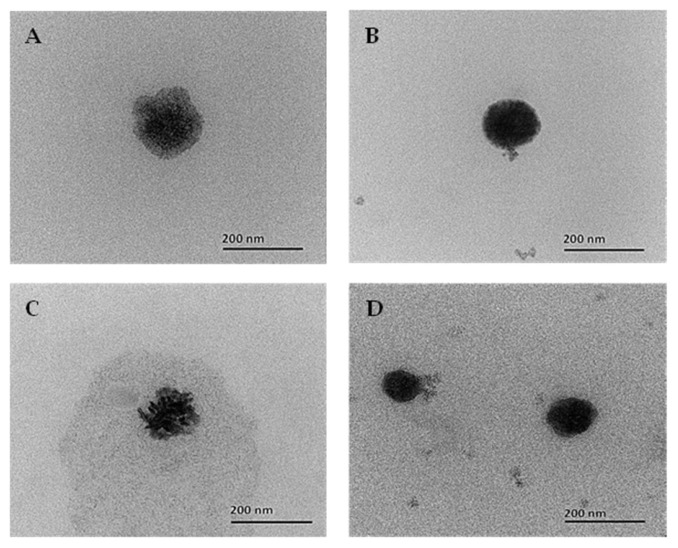
Transmission electron microscopy (TEM) images showing morphology of nanostructured lipid carrier formulations. The OE-L^+^NLCs (**A**) and L^+^NLCs (**C**) displayed nearly spherical and uniformly distributed nanoparticles surrounded by a thin lipid layer, while the OE-NLCs (**B**) and blank NLCs (**D**) showed smooth spherical structures. Scale bar = 200 nm.

TEM revealed that the OE-L^+^NLCs (Figure 1A) and L^+^NLCs (Figure 1C) were nearly spherical and uniformly distributed, with smooth surfaces surrounded by a thin lipid–surfactant layer. The OE-NLCs (Figure 1B) and blank NLCs (Figure 1D) showed smooth spherical structures. The morphology and size observed under TEM corresponded closely with the DLS data, confirming successful nanoparticle formation below 200 nm.

### 3.2. Encapsulation Efficiency, Drug Loading, and In Vitro Release of Oregano Oil

The EE and DL of carvacrol in the oregano oil-loaded nanostructured lipid carriers are presented in Table 2. The OE-L^+^NLCs formulation exhibited significantly higher EE (84.84 ± 1.51%) and DL (17.18 ± 0.50%) values compared with OE-NLCs, which showed EE and DL values of 76.67 ± 2.55% and 14.28 ± 0.83%, respectively (*p* < 0.05). These findings suggest that incorporation of lauric acid improved the retention and loading capacity of carvacrol within the lipid nanocarrier system.

**Figure 2:**
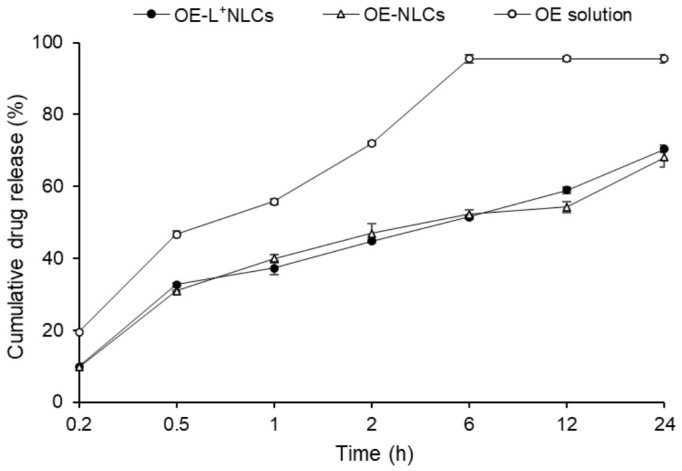
In vitro release profiles of carvacrol from oregano oil–lauric acid nanostructured lipid carriers (OE-L^+^NLCs), oregano oil nanostructured lipid carriers (OE-NLCs), and free oregano oil (OE) solution in phosphate-buffered saline (PBS, pH 7.4) containing 0.5% Tween 80 at 37 °C (*n* = 3). Encapsulation reduced burst release and provided sustained release over 24 h compared with rapid release from the free OE solution.

The release profile of carvacrol from OE-L^+^NLCs, OE-NLCs, and free OE solution demonstrated two distinct phases: an initial burst release within the first 6 h, followed by a sustained release phase over 24 h (Figure 2). After 24 h, the cumulative carvacrol release from OE-L^+^NLCs and OE-NLCs reached 70.46 ± 0.96% and 68.10 ± 2.74%, respectively, whereas the OE solution released 95.54 ± 1.18% within only 6 h. The controlled release observed in the NLC formulations suggests that encapsulation effectively reduced volatility and prolonged bioavailability.

### 3.3. Antibacterial Activity Against Isolates of S. agalactiae

All nanoparticle formulations exhibited measurable antibacterial activity against six *S. agalactiae* isolates from diseased Nile tilapia. The resulting MIC and MBC values are summarized in Table 3. The OE-L^+^NLCs and L^+^NLCs showed the strongest inhibition values, with an MIC of 0.25 mg/mL and an MBC of 0.25–0.49 mg/mL, consistent across all isolates. The L^+^ solution exhibited comparable activity, confirming the potent antibacterial contribution of lauric acid.

In contrast, the OE-NLCs and the free OE solution showed higher MIC values (7.81 mg/mL) than the cationic formulations. Blank NLCs showed no inhibitory activity, confirming that the antibacterial effect originated from the active components rather than the carrier matrix. The greater efficacy of OE-L^+^NLCs suggests a synergistic interaction between OE and cationic L^+^, enhancing bacterial membrane disruption and prolonging antibacterial activity.

### 3.4. Thermal Stability

#### 3.4.1. Physical and Physicochemical Characterizations

**Figure 3:**
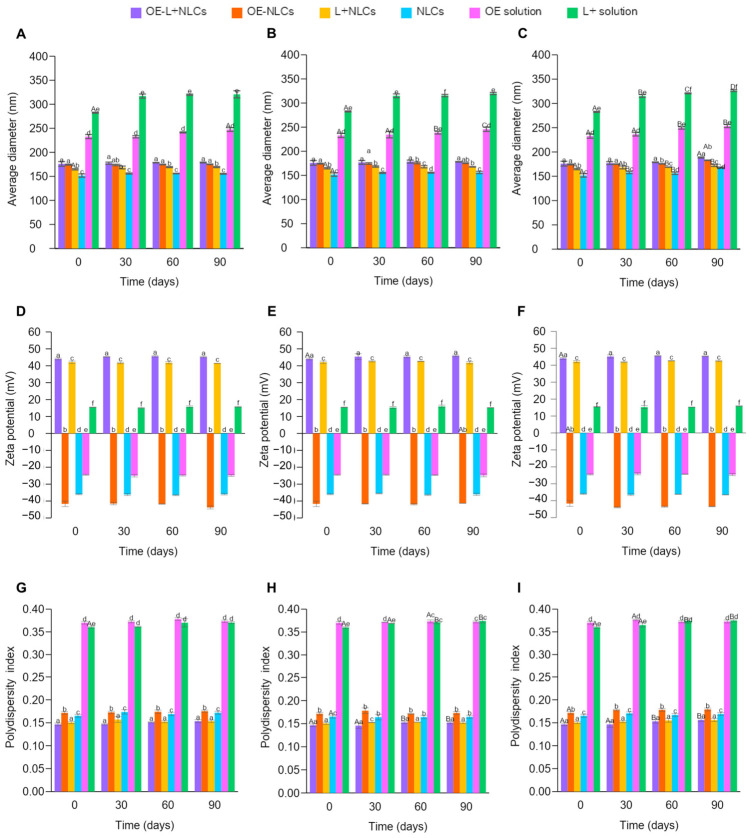
Physicochemical stability of nanostructured lipid carrier (NLC) formulations during 90 days of storage at 4 °C, 25 °C, and 37 °C. (**A**–**C**) Average particle diameter, (**D**–**F**) zeta potential, and (**G**–**I**) polydispersity index (PDI) of oregano oil–lauric acid cationic NLCs (OE-L^+^NLCs), oregano oil NLCs (OE-NLCs), lauric acid NLCs (L^+^NLCs), blank NLCs, oregano oil (OE) solution, and lauric acid (L^+^) solution were determined using dynamic light scattering over 0, 30, 60, and 90 days. All NLC formulations-maintained nanoscale size (<200 nm), narrow distribution (PDI < 0.3), and stable surface charge throughout the storage period. OE-L^+^NLCs and L^+^NLCs exhibited the highest stability, while unencapsulated OE and L^+^ solutions showed marked increases in particle size and heterogeneity, particularly at 37 °C. Different lowercase letters (a–f) within each time point indicate significant differences among formulations, while different uppercase letters (A–D) denote significant differences among time points.

All formulations remained stable with no visual phase separation or crystallization after over 90 days at 4 °C, 25 °C, and 37 °C. Only the L^+^ solution exhibited crystallization at 4 °C, indicating poor cold-storage stability (Figure S5). DLS measurements confirmed the strong physicochemical stability of all NLC formulations under different storage temperatures (4 °C, 25 °C, and 37 °C) for 90 days (Figure 3). All NLCs retained their nanoscale particle size (<200 nm) and low PDI values (<0.3), indicating uniform dispersion and absence of aggregation. Both OE-L^+^NLCs and L^+^NLCs maintained a positive surface charge (>+40 mV), ensuring good colloidal stability throughout the study period.

After 90 days at 37 °C, OE-L^+^NLCs and OE-NLCs showed a slight but statistically significant increase in particle size (*p* < 0.05). However, this change did not affect dispersion or overall appearance. In contrast, the unencapsulated OE and L^+^ solutions were markedly less stable, with particle sizes exceeding 300 nm, lower surface charge magnitude, and high PDI values (>0.35), indicating aggregation and poor thermal stability.

#### 3.4.2. Antibacterial Activity

The antibacterial activity of OE-L^+^NLCs, OE-NLCs, L^+^NLCs, NLCs, the OE solution and the L^+^ solution against *S. agalactiae* strain AY19 was evaluated after 0, 30, 60, and 90 days of storage at 25 °C, 4 °C, and 37 °C, as shown in Table 4. OE- L^+^ NLCs, L^+^ NLCs, and the L^+^ solution maintained stable MIC and MBC values of 0.25 mg/mL and 0.49 mg/mL respectively, across all time points and temperatures, indicating consistent antibacterial activity. OE-NLCs also remained stable under most conditions with MIC and MBC values of 7.81 mg/mL and 15.62 mg/mL, respectively, however, after 90 days at 37 °C these values increased to 15.62 mg/mL and >31.24 mg/mL, respectively. In contrast, the OE solution initially showed MBC values of 15.62 mg/mL but lost its antibacterial activity after 60 days at 37 °C. NLCs exhibited no antibacterial activity, confirming that the observed effects were due to the active components rather than the NLCs.

### 3.5. In Vitro Digestion

#### 3.5.1. Physical and Physicochemical Properties

The NLC formulations showed excellent stability under SGF conditions (pH 2.0), retaining their homogeneous appearance without phase separation (Figure S6). However, under SIF conditions (pH 7.0), OE-L^+^NLCs, OE-NLCs, and L^+^NLCs exhibited mild color changes and precipitation, indicating partial destabilization of the particles. Physicochemical measurements showed that, under SGF, NLC particle size and PDI remained below 200 nm and 0.3, respectively, indicating stability under acidic conditions. In contrast, exposure to SIF increased particle size and PDI (>0.3), suggesting reduced colloidal stability, likely due to bile salt-induced aggregation (Table 5). Unencapsulated OE and L^+^ solutions were markedly less stable, with particle sizes exceeding 300 nm and fluctuating zeta potentials across both digestive conditions.

#### 3.5.2. Antibacterial Activity

After gastric digestion, OE-L^+^NLCs and L^+^NLCs retained strong antibacterial activity, with MIC and MBC values of 0.25 mg/mL and 0.49 mg/mL, respectively, comparable to pre-digestion levels. OE-NLCs also remained active, showing an MIC of 7.81 mg/mL, whereas the OE solution lost most of its antibacterial effect under SGF. Under intestinal conditions, all formulations, including OE-L^+^NLCs, lost antibacterial activity (MIC > 31.24 mg/mL), likely due to lipid carrier degradation and the release of active compounds into micellar forms (Table 6).

### 3.6. Acute Toxicity Assessment Based on LC_50_ Values

The LC_50_ values of the tested formulations ranged from 5.89 to 12.48 mg/mL, indicating differences in toxicity among treatments (Table 7). The OE solution had the lowest LC_50_ value (5.89 mg/mL), followed by L^+^ solution (8.57 mg/mL). In contrast, the NLC-based formulations showed higher LC_50_ values, with L^+^NLCs recording the highest value (12.48 mg/mL), followed by OE-L^+^NLCs (11.11 mg/mL) and OE-NLCs (10.26 mg/mL). Probit analysis also showed variation in slope values among formulations. OE-L^+^NLCs had the highest slope (3.66 ± 0.44), whereas L^+^NLCs had the lowest (2.05 ± 0.21). Intermediate slopes were observed for OE-NLCs (2.92 ± 0.32), the OE solution (2.87 ± 0.34), and the L^+^ solution (2.35 ± 0.25).

### 3.7. Survival Analysis of Nile Tilapia Fed Different Formulations

**Figure 4:**
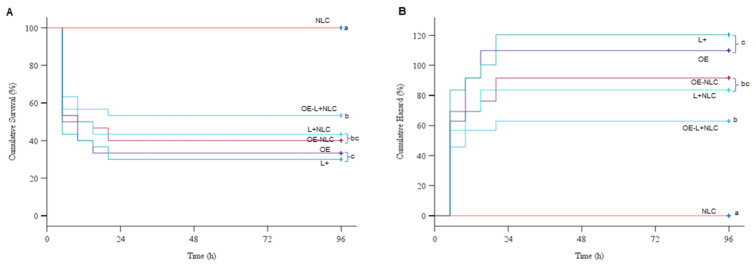
Kaplan–Meier survival and cumulative hazard analysis of Nile tilapia exposed to different formulations during acute toxicity assessment. (**A**) Kaplan–Meier survival curves showing cumulative survival (%) over 96 h. (**B**) Corresponding cumulative hazard curves over time. Treatments include OE-L^+^NLCs, OE-NLCs, L^+^NLCs, OE solution, L^+^ solution, and blank NLCs. Different superscript letters indicate statistically significant differences among treatments (log-rank test, *p* < 0.05).

Kaplan–Meier survival analysis showed differences in survival probability among treatment groups over the 96 h feeding period (Figure 4A). The cumulative survival curves indicated that the NLC group maintained the highest survival (~100%), while lower survival rates were observed in the L^+^ and OE groups. The OE-NLC, L^+^NLC, and OE-L^+^NLC groups showed intermediate survival patterns. The log-rank (Mantel–Cox) test revealed significant differences in survival distributions among the treatment groups (χ^2^ = 35.594, df = 5, *p* < 0.001).

Cox proportional hazards analysis demonstrated a significant overall effect of treatment on survival time (χ^2^ = 41.804, df = 5, *p* < 0.001) (Figure 4B). The estimated hazard ratios (Exp(B)) ranged from 1.262 to 1.637 across treatments. The OE-L^+^NLC group showed a hazard ratio approaching zero, whereas other treatments exhibited higher hazard ratio estimates. No individual treatment effects were statistically significant (*p* > 0.05). These survival patterns were consistent with the LC_50_ values, where formulations with higher LC_50_ exhibited higher survival probabilities.

### 3.8. Histopathological Evaluation of Dietary Toxicity in Nile Tilapia

**Figure 5:**
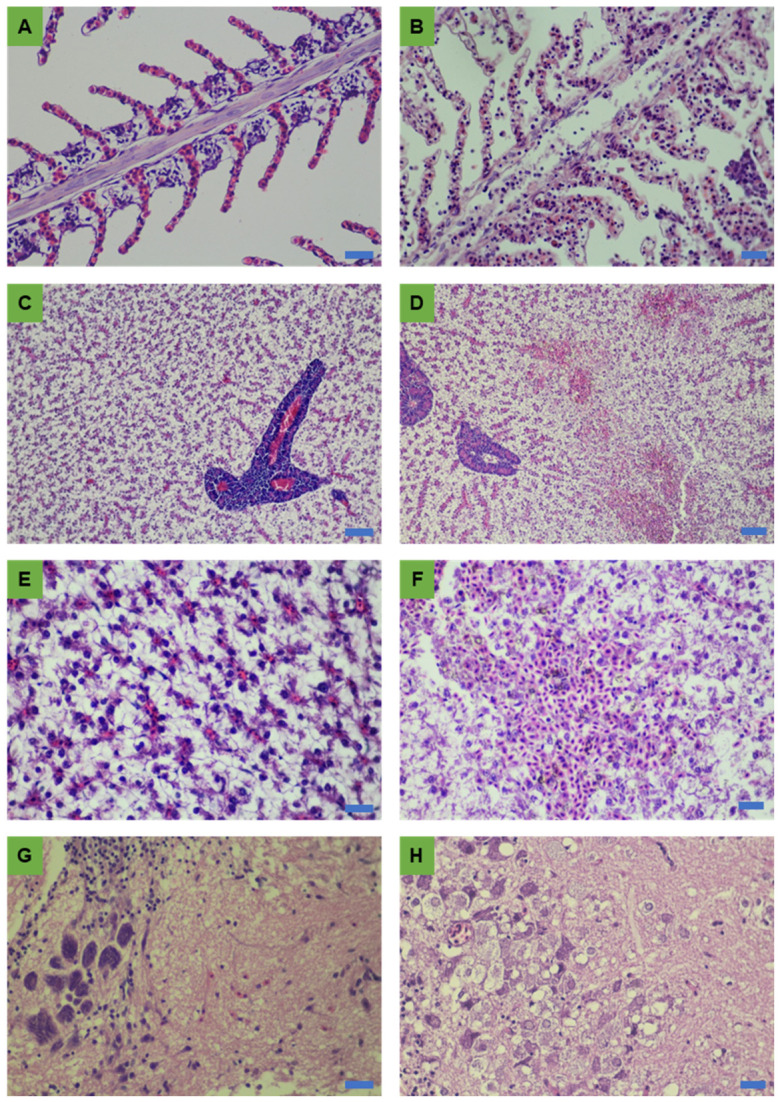
Histopathological changes in gill, liver, and brain tissues of Nile tilapia following dietary administration at LC_50_ concentrations compared with control fish. Normal gill architecture in control fish showing intact primary and secondary lamellae ((**A**); bar = 25 µm). Fish receiving LC_50_ dietary treatment showed severe edema and vascular congestion in the primary and secondary lamellae with dissociation of the pillar capillary system ((**B**); bar = 25 µm). Normal liver and hepatopancreas architecture in control fish ((**C**); bar = 50 µm; (**E**); bar = 25 µm). Liver tissue of LC_50_-treated fish showing multifocal hemorrhages in the parenchyma ((**D**); bar = 25 µm) and hepatocellular swelling with dissociation of hepatic cords ((**F**); bar = 25 µm). Normal brain histology in control fish ((**G**); bar = 25 µm). Brain tissue of LC_50_-treated fish showing severe neuronal edema with cytoplasmic vacuolation and vacuolar degeneration of the neuropil ((**H**); bar = 25 µm).

Histopathological examination revealed clear alterations in the gill, liver, and brain tissues of Nile tilapia following dietary administration at LC_50_ concentrations, compared with control fish (Figure 5). In the gills, control fish showed normal architecture with intact primary and secondary lamellae (Figure 5A), whereas treated fish exhibited marked edema, vascular congestion, and dissociation of the pillar capillary system in both primary and secondary lamellae (Figure 5B). In the liver, control fish displayed normal hepatopancreatic architecture (Figure 5C,E).

Fish receiving the LC_50_ dietary treatment showed multifocal hemorrhages in the hepatic parenchyma (Figure 3D), together with hepatocellular swelling and disruption of hepatic cord architecture (Figure 3F). Normal histological structure was observed in the brain of control fish (Figure 3G), whereas treated fish showed severe neuronal edema, cytoplasmic vacuolation, and vacuolar degeneration of the neuropil (Figure 3H). These histopathological alterations were consistent with the observed toxicity patterns, with formulations associated with lower LC_50_ values and lower survival causing more severe tissue damage.

## 4. Discussion

The physicochemical results indicate that the formulated NLCs have properties well suited for delivering bioactive compounds in aquaculture. Their nanoscale particle size is a key factor influencing biological interactions, mucosal contact, and the functional efficiency of feed additives in aquatic species [61]. Similar particle sizes have been reported for other essential oil-loaded lipid nanocarriers, including clove oil-based NLCs, supporting the suitability of the present formulation approach [62]. Surface charge was another important distinguishing feature. Incorporation of cationic lauric acid produced a strong positive surface charge, which improves colloidal stability through electrostatic repulsion and facilitates interaction with negatively charged bacterial membranes and fish mucosal surfaces [63,64]. This cationic behavior has been linked with improved antimicrobial activity and mucosal adhesion in lipid-based delivery systems [65,66]. By contrast, non-cationic essential oil-loaded NLCs usually carry an anionic surface charge, which may reduce membrane interaction [62,67]. The uniform spherical morphology observed further indicates optimized lipid–surfactant assembly, which is important for maintaining encapsulation efficiency and limiting premature release of volatile compounds [68]. Collectively, these findings show that nanoencapsulation improves the physicochemical stability of oregano oil, while the inclusion of lauric acid further enhances properties associated with antimicrobial performance, consistent with previous reports on the synergistic benefits of lipid-based nanocarriers for essential oils [69,70,71].

The sustained release behavior of carvacrol from OE-L^+^NLCs and OE-NLCs, compared with free OE reflects effective entrapment of the volatile compound within the lipid matrix. Consistent with this observation, OE-L^+^NLCs exhibited higher encapsulation efficiency and drug loading than OE-NLCs, suggesting that incorporation of lauric acid improved the retention capacity of the lipid system for hydrophobic bioactive compounds. The enhanced encapsulation performance may also have contributed to the prolonged release behavior by reducing premature leakage of oregano oil constituents during nanoparticle formation and storage. The observed release profile is characteristic of diffusion-controlled systems, where an initial release of surface-associated carvacrol is followed by gradual diffusion from the lipid core over time [72,73,74]. Similar release kinetics and encapsulation improvements have been reported for essential oil-loaded NLCs and lipid-based nanocarriers containing hydrophobic phytochemicals, where nanoencapsulation delayed release and improved physicochemical stability relative to free compounds [62,75,76,77,78]. For aquaculture feed applications, this controlled release is especially beneficial because it prolongs the availability of bioactive compounds, supports formulation stability, and helps explain the sustained antibacterial activity observed.

The strong antibacterial activity exhibited by OE-L^+^NLCs and L^+^NLCs highlights the central role of cationic lauric acid in inhibiting *S. agalactiae*. Their performance, comparable to free L^+^, is consistent with previous studies identifying L^+^ as a potent antimicrobial agent against Gram-positive fish pathogens [79]. The strong antibacterial activity exhibited by OE-L^+^NLCs was comparable to that of L^+^NLCs and free L^+^ solution, indicating that the direct antibacterial effect against *S. agalactiae* was primarily associated with the lauric acid component under the present in vitro conditions, while oregano oil may contribute to formulation stability, sustained release behavior, and lipid phase interactions within the nanocarrier system despite no additional measurable reduction in MIC/MBC values. Mechanistically, the cationic surface charge promotes electrostatic interaction with negatively charged bacterial membranes, facilitating membrane destabilization, while L^+^ disrupts membrane integrity, interferes with electron transport, and inhibits membrane-associated enzymes, ultimately leading to bacterial cell lysis [70,80]. Nanoencapsulation may further contribute by stabilizing both L^+^ and OE and prolonging the bioavailability of hydrophobic compounds relative to non-encapsulated forms [81,82]. These findings align with earlier reports demonstrating strong antibacterial activity of L^+^ against *S. agalactiae* [79] and broad-spectrum antimicrobial effects of OE rich in carvacrol and thymol against *Streptococcus* species [83,84]. The lack of activity in blank NLCs confirms that the antibacterial effects were due to the active compounds rather than the carrier matrix. Based on this initial screening, strain AY19 was chosen as the representative isolate for the thermal stability and digestion studies because its susceptibility pattern was similar to those of the other isolates, enabling a focused assessment of formulation stability and bioactivity without unnecessary duplication.

The NLC formulations demonstrated excellent thermal stability over prolonged storage at refrigeration, room, and physiological temperatures, as evidenced by their maintained nanoscale particle size, low PDI, and stable surface charge. In contrast, crystallization of the unencapsulated L^+^ solution under cold storage highlights the greater temperature sensitivity of free compounds. The strong positive surface charge retained by OE-L^+^NLCs and L^+^NLCs further supports their robust colloidal stability, promoting electrostatic repulsion [73] and interaction with negatively charged bacterial membranes [85,86]. Although particle size increased slightly at higher temperatures, formulation integrity was not compromised. These findings suggest that the NLC matrix protects OE and L^+^ from thermal stress [87,88,89], thereby reducing aggregation, volatilization, and degradation commonly seen in unencapsulated systems [90,91]. Similar stabilization has been reported for other lipid-based nanocarriers, including curcumin- and essential oil-loaded NLCs, where the lipid matrix provided long-term physicochemical protection during storage [74,81]. Importantly, the preserved physicochemical stability translated directly into sustained antibacterial efficacy. OE-L^+^NLCs and L^+^NLCs maintained consistent antibacterial activity across all storage conditions, whereas free OE lost activity rapidly at elevated temperatures. Comparable preservation of antimicrobial function has been reported for nanoencapsulated OE and other essential oils under environmental stress conditions [84]. Collectively, these findings support the use of NLCs as effective carriers for thermolabile antimicrobial agents, ensuring long-term stability and functional reliability in aquaculture feed applications [82].

The NLC formulations exhibited high stability under simulated gastric conditions, maintaining homogeneous appearance, nanoscale particle size, and low PDI, thereby preserving both nanoparticle integrity and bioactivity in an acidic environment [92]. This gastric stability is essential for oral delivery in fish, as it ensures protection of encapsulated OE and L^+^ during stomach passage [90,93]. In contrast, unencapsulated formulations showed pronounced instability, highlighting the protective role of the NLC matrix. Upon exposure to simulated intestinal conditions, all NLC formulations showed partial destabilization, characterized by increased particle size, elevated PDI, and visible structural changes. This behavior is likely driven by the combined effects of elevated pH, bile salts, and digestive enzymes, which disrupt lipid–surfactant interfaces and promote nanoparticle restructuring or aggregation [94,95,96]. Comparable digestion-dependent behavior has been observed in other lipid-based and essential oil-loaded nanocarriers, which are generally stable under gastric conditions but undergo structural rearrangements in the intestinal environment [97,98]. Consistent with these physicochemical changes, OE-L^+^NLCs and L^+^NLCs retained strong antibacterial activity after gastric digestion, confirming that nanoencapsulation effectively protects active compounds from acidic degradation [92,99]. However, antibacterial activity was markedly reduced under simulated intestinal conditions, likely due to carrier destabilization [97,100], rapid release of the bioactives [101], and the complex intestinal environment (including bile salts, digestive enzymes, ionic strength, and pH changes), which may alter the nanoparticle integrity, weaken electrostatic interactions, and reduce the sustained antibacterial functionality of the intact nanocarrier system [100]. Similar digestion-related reductions in antimicrobial efficacy have been reported for essential oil-loaded nanoparticles [31], where sustained activity throughout the intestinal phase often requires further formulation optimization [102,103].

Building on these in vitro findings, the in vivo toxicity assessment provides important insight into the biological safety of the formulations. LC_50_ values (5.89–12.48 mg/mL) revealed marked formulation-dependent differences, with NLC-based formulations showing lower toxicity than non-encapsulated forms. The OE solution had the lowest LC_50_ (5.89 mg/mL), whereas L^+^NLCs showed the highest (12.48 mg/mL), demonstrating that encapsulation substantially improved safety [104]. This improved safety profile is consistent with the controlled release behavior and physicochemical stability observed in vitro, as nanoencapsulation reduces sudden peak exposure to active compounds and enables more gradual and controlled release, thereby minimizing acute toxic effects [105]. The biocompatible lipid composition of NLCs may further enhance safety compared with other nanoparticle systems [93], while also improving the stability and delivery efficiency of hydrophobic phytochemicals such as essential oils [105,106,107]. Similar benefits have been reported for encapsulation approaches that improve solubility, reduce leaching, and enhance compound retention in aquatic environments [108].

The Kaplan–Meier survival analysis and Cox regression results further supported these findings, demonstrating that NLC formulations maintained higher survival probabilities compared with non-encapsulated treatments. Significant differences among treatment groups, confirmed by the log-rank test and Cox model, indicate that formulation significantly influenced mortality risk. The consistency between LC_50_ values and survival outcomes reinforces the reliability of the toxicity assessment, as higher LC_50_ values correspond to lower acute toxicity and reduced mortality risk [104]. This agreement highlights the importance of formulation design in determining not only efficacy but also safety in aquaculture applications.

Histopathological observations provided additional evidence supporting the improved safety of the NLC formulations. Gill lesions, including edema, vascular congestion, and pillar capillary dissociation, were more pronounced in non-encapsulated treatments and are well-recognized indicators of toxicant exposure [109,110]. The liver exhibited multifocal hemorrhages, hepatocellular swelling, and disruption of hepatic cord organization, consistent with tissue injury reported in fish exposed to high doses of essential oils [111,112]. In contrast, reduced hepatic damage in NLC-treated fish suggests that encapsulation moderated tissue distribution and accumulation, as previously observed for lipid-based nanocarriers [38,113]. The most severe changes were observed in the brain, where neuronal edema, cytoplasmic vacuolation, and neuropil degeneration were evident. Given the limited regenerative capacity of neural tissue, these findings are of particular toxicological concern. However, the reduced severity of brain lesions in NLC-treated groups suggests a potential neuroprotective effect, possibly due to controlled release and reduced systemic exposure to toxic metabolites [113,114,115]. The overall agreement among LC_50_ values, survival data, and histopathological findings represents a major strength of the study and supports the conclusion that nanoencapsulation improves the therapeutic index of bioactive compounds in fish [38,104,106,113].

These findings collectively indicate that nanoencapsulation enhances the stability, antibacterial efficacy, and safety profile of OE and L^+^, primarily through controlled release behavior and improved physicochemical protection. While OE-L^+^NLCs demonstrated strong performance under gastric conditions, the reduced antibacterial activity observed in simulated intestinal environments highlights a key limitation that requires further formulation optimization. In addition, as the present study was limited to acute toxicity assessment, future studies should focus on chronic exposure, tissue distribution, elimination kinetics, and long-term safety under practical aquaculture conditions.

## 5. Conclusions

OE-L^+^NLCs demonstrated strong in vitro suppression of *S. agalactiae*, coupled with controlled carvacrol release and excellent physicochemical stability during prolonged storage. The cationic nanoformulation preserved antibacterial performance under simulated gastric conditions, highlighting its suitability for oral, feed-based delivery in tilapia culture. In contrast, reduced stability and activity under intestinal simulation indicate a key area for further formulation optimization. Importantly, in vivo toxicity assessment showed that NLC-based formulations had higher LC_50_ values than non-encapsulated forms, indicating reduced acute toxicity and improved safety. Kaplan–Meier and Cox analyses confirmed higher survival and lower mortality risk in nanoencapsulated treatments. Histopathological findings further supported these results, showing reduced tissue damage in NLC-treated fish and suggesting improved biological tolerance through encapsulation. Overall, OE-L^+^NLCs represent a stable and effective nano-delivery platform for natural antimicrobials, combining antibacterial efficacy with improved acute safety, and therefore provide a promising antibiotic-alternative strategy that warrants future long-term in vivo efficacy and safety validation in aquaculture systems.

## Figures and Tables

**Table 1:** Physicochemical characteristics of oregano oil–lauric acid cationic nanostructured lipid carriers (OE-L^+^NLCs) and control formulations measured 24 h after preparation.

Formulations	Average Diameter (nm)	Zeta Potential (mV)	Polydispersity Index
OE-L^+^NLCs	175.90 ± 4.94 ^a^	+44.02 ± 0.54 ^a^	0.15 ± 0.00 ^a^
OE-NLCs	174.33 ± 0.92 ^a^	−41.69 ± 1.62 ^b^	0.17 ± 0.00 ^b^
L^+^NLCs	164.83 ± 1.44 ^b^	+42.12 ± 0.66 ^c^	0.15 ± 0.00 ^a^
NLCs	151.67 ± 3.51 ^c^	−35.95 ± 0.32 ^d^	0.17 ± 0.00 ^c^
OE solution	232.78 ± 4.31 ^d^	−24.50 ± 0.32 ^e^	0.37 ± 0.00 ^d^
L^+^ solution	282.50 ± 1.05 ^e^	+15.46 ± 0.15 ^f^	0.36 ± 0.00 ^e^

Results are expressed as mean ± SD (*n* = 3). Different superscript letters within each column indicate statistically significant differences (*p* < 0.05).

**Table 2:** Encapsulation efficiency (EE) and drug loading (DL) of carvacrol in oregano oil-loaded nanostructured lipid carrier formulations.

Formulations	Encapsulation Efficiency (%)	Drug Loading (%)
OE-L^+^NLCs	84.84 ± 1.51 ^a^	17.18 ± 0.50 ^a^
OE-NLCs	76.67 ± 2.55 ^b^	14.28 ± 0.83 ^b^

Results are expressed as mean ± SD (*n* = 3). Different superscript letters within the same column indicate statistically significant differences between formulations (Independent samples *t*-test, *p* < 0.05). OE-L^+^NLCs: oregano oil–lauric acid cationic nanostructured lipid carriers; OE-NLCs: oregano oil-loaded nanostructured lipid carriers.

**Table 3:** Minimum inhibitory concentration (MIC, mg/mL) and minimum bactericidal concentration (MBC, mg/mL) of nanostructured lipid carrier (NLC) formulations and control solutions against six *Streptococcus agalactiae* isolates from diseased Nile tilapia (*Oreochromis niloticus*).

Formulations	PC08		SM81		CT59		SM86		SM24		AY19	
	MIC	MBC	MIC	MBC	MIC	MBC	MIC	MBC	MIC	MBC	MIC	MBC
OE-L^+^NLCs	0.25	0.49	0.25	0.25	0.25	0.25	0.25	0.25	0.25	0.25	0.25	0.49
OE-NLCs	7.81	15.62	7.81	15.62	7.81	15.62	7.81	15.62	7.81	15.62	7.81	15.62
L^+^NLCs	0.25	0.49	0.25	0.25	0.25	0.25	0.25	0.25	0.25	0.25	0.25	0.49
NLCs	>31.25	>31.25	>31.25	>31.25	>31.25	>31.25	>31.25	>31.25	>31.25	>31.25	>31.25	>31.25
OE solution	7.81	15.62	7.81	15.62	7.81	15.62	7.81	15.62	7.81	15.62	7.81	15.62
L^+^ solution	0.25	0.49	0.25	0.25	0.25	0.25	0.25	0.25	0.25	0.25	0.25	0.49
ENR	7.81	15.62	3.91	7.81	3.91	7.81	3.91	7.81	1.95	3.91	7.81	15.62
DMSO	>31.25	>31.25	>31.25	>31.25	>31.25	>31.25	>31.25	>31.25	>31.25	>31.25	>31.25	>31.25

OE-L^+^NLCs: oregano oil-loaded lauric acid cationic nanostructured lipid carrier; OE-NLCs: oregano oil-loaded nanostructured lipid carrier; L^+^NLC: lauric acid cationic nanostructured lipid carrier; NLCs: nanostructured lipid carrier blank; OE solution was dissolved in 99.9% (*v*/*v*) DMSO; L^+^ solution was dissolved in distilled water, ENR: enrofloxacin (5 µg/mL) and DMSO: dimethyl sulfoxide.

**Table 4:** Minimum inhibitory concentration (MIC, mg/mL) and minimum bactericidal concentration (MBC, mg/mL) values of the formulations under thermal stability experiments (25 °C, 4 °C, and 37 °C) at 0, 30, 60, and 90 days.

Formulations	Antibacterial	25 °C				4 °C				37 °C			
		0 d	30 d	60 d	90 d	0 d	30 d	60 d	90 d	0 d	30 d	60 d	90 d
OE-L^+^NLCs	MIC	0.25	0.25	0.25	0.25	0.25	0.25	0.25	0.25	0.25	0.25	0.25	0.25
	MBC	0.49	0.49	0.49	0.49	0.49	0.49	0.49	0.49	0.49	0.49	0.49	0.49
OE-NLCs	MIC	7.81	7.81	7.81	7.81	7.81	7.81	7.81	7.81	7.81	7.81	7.81	15.62
	MBC	15.62	15.62	15.62	15.62	15.62	15.62	15.62	15.62	15.62	15.62	15.62	>31.24
L^+^NLCs	MIC	0.25	0.25	0.25	0.25	0.25	0.25	0.25	0.25	0.25	0.25	0.25	0.25
	MBC	0.49	0.49	0.49	0.49	0.49	0.49	0.49	0.49	0.49	0.49	0.49	0.49
NLCs	MIC	>31.24	>31.24	>31.24	>31.24	>31.24	>31.24	>31.24	>31.24	>31.24	>31.24	>31.24	>31.24
	MBC	>31.24	>31.24	>31.24	>31.24	>31.24	>31.24	>31.24	>31.24	>31.24	>31.24	>31.24	>31.24
OE solution	MIC	7.81	7.81	7.81	7.81	7.81	7.81	7.81	7.81	7.81	7.81	15.62	15.62
	MBC	15.62	15.62	15.62	15.62	15.62	15.62	15.62	15.62	15.62	15.62	>31.24	>31.24
L^+^ solution	MIC	0.25	0.25	0.25	0.25	0.25	0.25	0.25	0.25	0.25	0.25	0.25	0.25
	MBC	0.49	0.49	0.49	0.49	0.49	0.49	0.49	0.49	0.49	0.49	0.49	0.49

OE-L^+^NLCs: oregano oil-loaded lauric acid cationic nanostructured lipid carrier; OE-NLCs: oregano oil-loaded nanostructured lipid carrier; L^+^NLC: lauric acid cationic nanostructured lipid carrier; NLCs: nanostructured lipid carrier blank; OE solution was dissolved in 99.9% (*v*/*v*) DMSO; L^+^ solution was dissolved in distilled water.

**Table 5:** In vitro stability of the preparations in simulated gastric fluid (SGF) and simulated intestinal fluid (SIF) to average diameter (nm), zeta potential (mV), polydispersity index, and antibacterial activity against *S. agalactiae* strain AY19.

Formulations	Average Diameter (nm)			Zeta Potential (mV)			Polydispersity Index		
	Normal	SGF	SIF	Normal	SGF	SIF	Normal	SGF	SIF
OE-L^+^NLCs	175.9 ± 4.9 ^Ac^	174.4 ± 2.5 ^Ab^	188.6 ± 3.0 ^Bc^	+44.0 ± 0.5 ^Af^	+43.1 ± 0.8 ^Af^	+54.6 ± 1.4 ^Be^	0.15 ± 0.00 ^Aa^	0.16 ± 0.00 ^Bb^	0.37 ± 0.00 ^Cb^
OE-NLCs	174.3 ± 0.9 ^Ac^	173.6 ± 3.0 ^Ab^	185.4 ± 2.7 ^Bbc^	−41.7 ± 1.6 ^Aa^	−41.6 ± 1.4 ^Aa^	−33.2 ± 1.7 ^Ba^	0.17 ± 0.00 ^Ac^	0.18 ± 0.00 ^Bc^	0.32 ± 0.00 ^Ca^
L^+^NLCs	164.8 ± 1.4 ^Ab^	165.7 ± 1.2 ^Aa^	181.0 ± 2.1 ^Cb^	+42.1 ± 0.7 ^Ae^	+40.5 ± 1.8 ^Ae^	+57.6 ± 1.5 ^Bf^	0.15 ± 0.00 ^Aa^	0.19 ± 0.00 ^Bc^	0.37 ± 0.00 ^Cc^
NLCs	151.7 ± 3.5 ^Aa^	152.6 ± 3.0 ^Aa^	163.7 ± 2.6 ^Ba^	−36.0 ± 0.3 ^Ab^	−37.4 ± 0.5 ^Ab^	−27.5 ± 1.3 ^Bb^	0.17 ± 0.00 ^Ab^	0.15 ± 0.00 ^Ba^	0.37 ± 0.00 ^Cb^
OE solution	232.8 ± 4.3 ^Ad^	255.0 ± 5.6 ^Bc^	325.2 ± 3.4 ^Cd^	−24.5 ± 0.3 ^Ac^	−22.7 ± 0.4 ^Bc^	−15.4 ± 0.6 ^Cc^	0.37 ± 0.00 ^Ae^	0.40 ± 0.00 ^Be^	0.41 ± 0.00 ^Cd^
L^+^ solution	282.5 ± 1.1 ^Ae^	283.0 ± 3.6 ^Ad^	378.5 ± 3.8 ^Ce^	+15.5 ± 0.2 ^Ad^	+17.5 ± 0.5 ^Ad^	+28.6 ± 2.0 ^Bd^	0.36 ± 0.00 ^Ad^	0.35 ± 0.00 ^Bd^	0.42 ± 0.00 ^Cd^

Results are mean ± SD (*n* = 3). Superscript uppercase letters indicate significant differences among digestive conditions (Normal, SGF, and SIF) for each parameter, while different lowercase letters indicate significant differences among treatments within the same column (*p* < 0.05). SGF: simulated gastric fluid, SIF: simulated intestinal fluid, OE-L^+^NLCs: oregano oil-loaded lauric acid cationic nanostructured lipid carrier; OE-NLCs: oregano oil-loaded nanostructured lipid carrier; L^+^NLC: lauric acid cationic nanostructured lipid carrier; NLCs: nanostructured lipid carrier blank; OE solution was dissolved in 0.5% (*v*/*v*) DMSO; L^+^ solution was dissolved in distilled water.

**Table 6:** In vitro stability of the preparations in simulated gastric fluid (SGF) and simulated intestinal fluid (SIF) to antibacterial activity (mg/mL) against *S. agalactiae* strain AY19.

Formulations	Normal		SGF		SIF	
	MIC	MBC	MIC	MBC	MIC	MBC
OE-L^+^NLCs	0.25	0.49	0.25	0.49	>31.24	>31.24
OE-NLCs	7.81	15.62	7.81	15.62	>31.24	>31.24
L^+^NLCs	0.25	0.49	0.25	0.49	>31.24	>31.24
NLCs	>31.24	>31.24	>31.24	>31.24	>31.24	>31.24
OE solution	7.81	15.62	>31.24	>31.24	>31.24	>31.24
L^+^ solution	0.25	0.49	0.25	0.49	>31.24	>31.24

MIC: minimum inhibitory concentration, MBC: minimum bactericidal, OE-L^+^NLCs: oregano oil-loaded lauric acid cationic nanostructured lipid carrier; OE-NLCs: oregano oil-loaded nanostructured lipid carrier; L^+^NLC: lauric acid cationic nanostructured lipid carrier; NLCs: nanostructured lipid carrier blank; OE solution was dissolved in 99.9% (*v*/*v*) DMSO; L^+^ solution was dissolved in distilled water.

**Table 7:** Acute oral toxicity (LC_50_) of different formulations in Nile tilapia (*Oreochromis niloticus*) following 96 h dietary administration.

Formulations	LC_50_ (mg/mL)	95% Confidence Interval	Slope ± SE
OE-L^+^NLCs	11.11	9.38–13.41	3.66 ± 0.44
OE-NLCs	10.26	8.54–12.58	2.92 ± 0.32
L^+^NLCs	12.48	9.95–15.92	2.05 ± 0.21
OE solution	5.89	4.95–7.10	2.87 ± 0.34
L^+^ solution	8.57	6.54–11.48	2.35 ± 0.25

## Data Availability

Data are contained within the article or Supplementary Material.

## Supplementary Materials

The following supporting information can be downloaded at: https://www.mdpi.com/article/10.3390/ani16111639/s1, Figure S1. Schematic representation of OE-L^+^NLCs preparation process. Stepwise illustration of the emulsification–sonication technique used to produce oregano oil–lauric acid cationic nanostructured lipid carriers; Figure S2. Representative microplate images of MIC and MBC assays. Visual confirmation of bacterial inhibition zones corresponding to the microdilution assay results for *S. agalactiae*; Figure S3. Flow diagram of the in vitro digestion procedure. Experimental setup showing simulated gastric (SGF) and intestinal (SIF) digestion steps for evaluating formulation stability and bioactivity; Figure S4. Physical appearance of oregano oil–lauric acid cationic nanostructured lipid carriers (OE-L^+^NLCs) and control formulations 24 h after preparation at 25 °C. (A) OE-L^+^NLCs, (B) OE-NLCs, (C) L^+^NLCs, (D) blank NLCs, (E) oregano oil (OE) solution, and (F) lauric acid (L^+^) solution. Formulations containing NLCs exhibited a uniform emulsion appearance without phase separation; Figure S5. Physical stability of oregano oil–lauric acid cationic nanostructured lipid carriers (OE-L^+^NLCs) and control formulations stored at 25 °C, 4 °C, and 37 °C for 90 days. All NLC formulations (OE-L^+^NLCs, OE-NLCs, L^+^NLCs, and blank NLCs) remained homogeneous without phase separation, whereas the L^+^ solution showed crystallization at 4 °C. The OE solution displayed color darkening but no solidification, indicating oxidation of free oil; Figure S6. Physical characterization of OE-L^+^NLCs, OE-NLCs, L^+^NLCs and NLCs in SGF and SIF; visual observation of samples after 2 h of digestion in SGF and SIF, respectively; Table S1. Detailed formulation composition of OE-L^+^NLCs, OE-NLCs, L^+^NLCs, and blank NLCs.
