# Supplementary material for: Antibacterial Activity and Safety of Oregano Oil–Lauric Acid Cationic Nanostructured Lipid Carriers in Nile Tilapia (Oreochromis niloticus)
Source: Animals (Basel). 2026 May 27;16(11):1639. doi: 10.3390/ani16111639 (PMC13255757; doi:10.3390/ani16111639)
Supplement: Supplementary file 1 [file animals-16-01639-s001.zip › Supplementary Figures_OEL+NLC_Animals.pptx]

## Slide 1
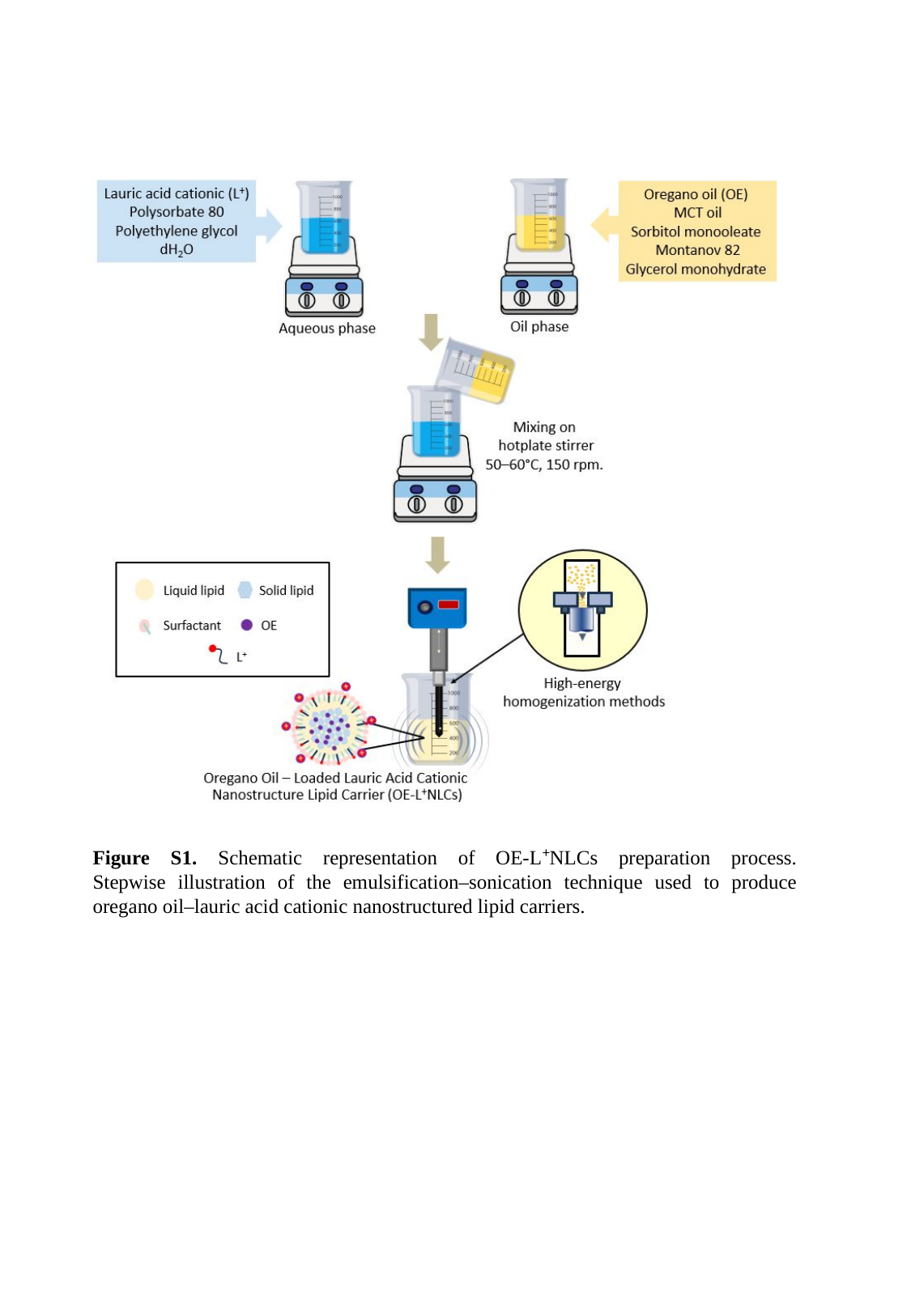

Figure S1. Schematic representation of OE-L⁺NLCs preparation process.
Stepwise illustration of the emulsification–sonication technique used to produce oregano oil–lauric acid cationic nanostructured lipid carriers.

## Slide 2
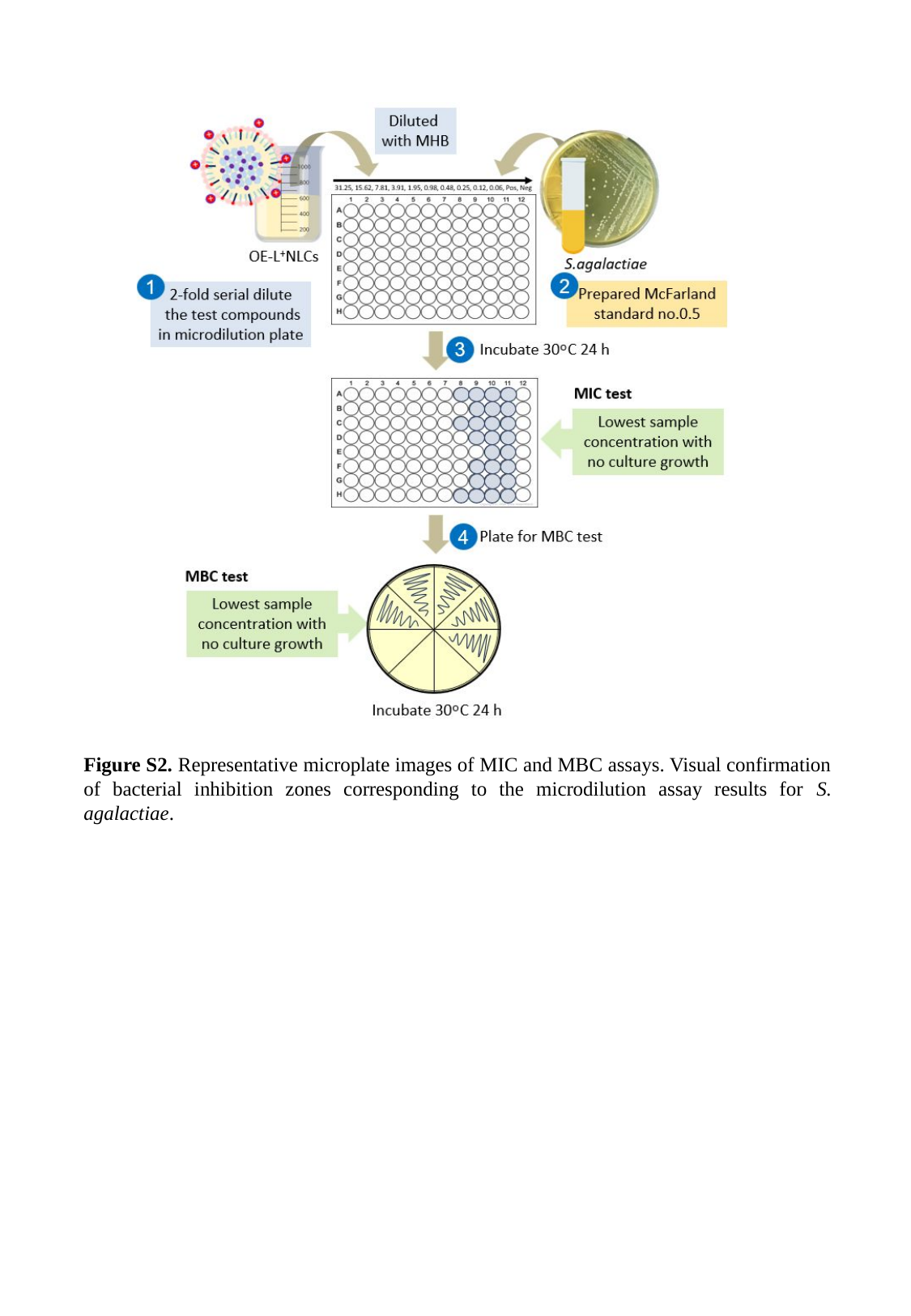

Figure S2. Representative microplate images of MIC and MBC assays. Visual confirmation of bacterial inhibition zones corresponding to the microdilution assay results for S. agalactiae.

## Slide 3
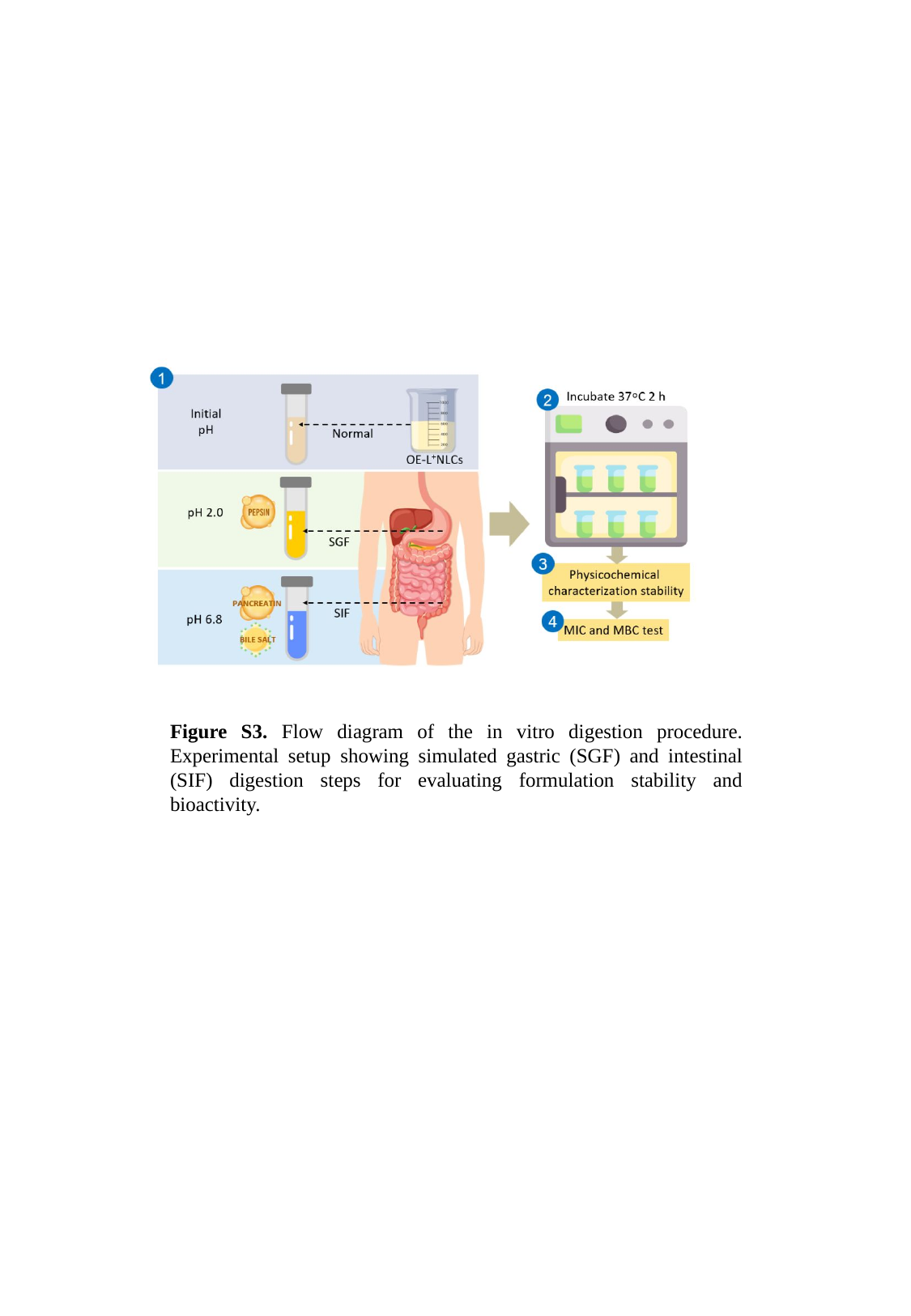

Figure S3. Flow diagram of the in vitro digestion procedure. Experimental setup showing simulated gastric (SGF) and intestinal (SIF) digestion steps for evaluating formulation stability and bioactivity.

## Slide 4
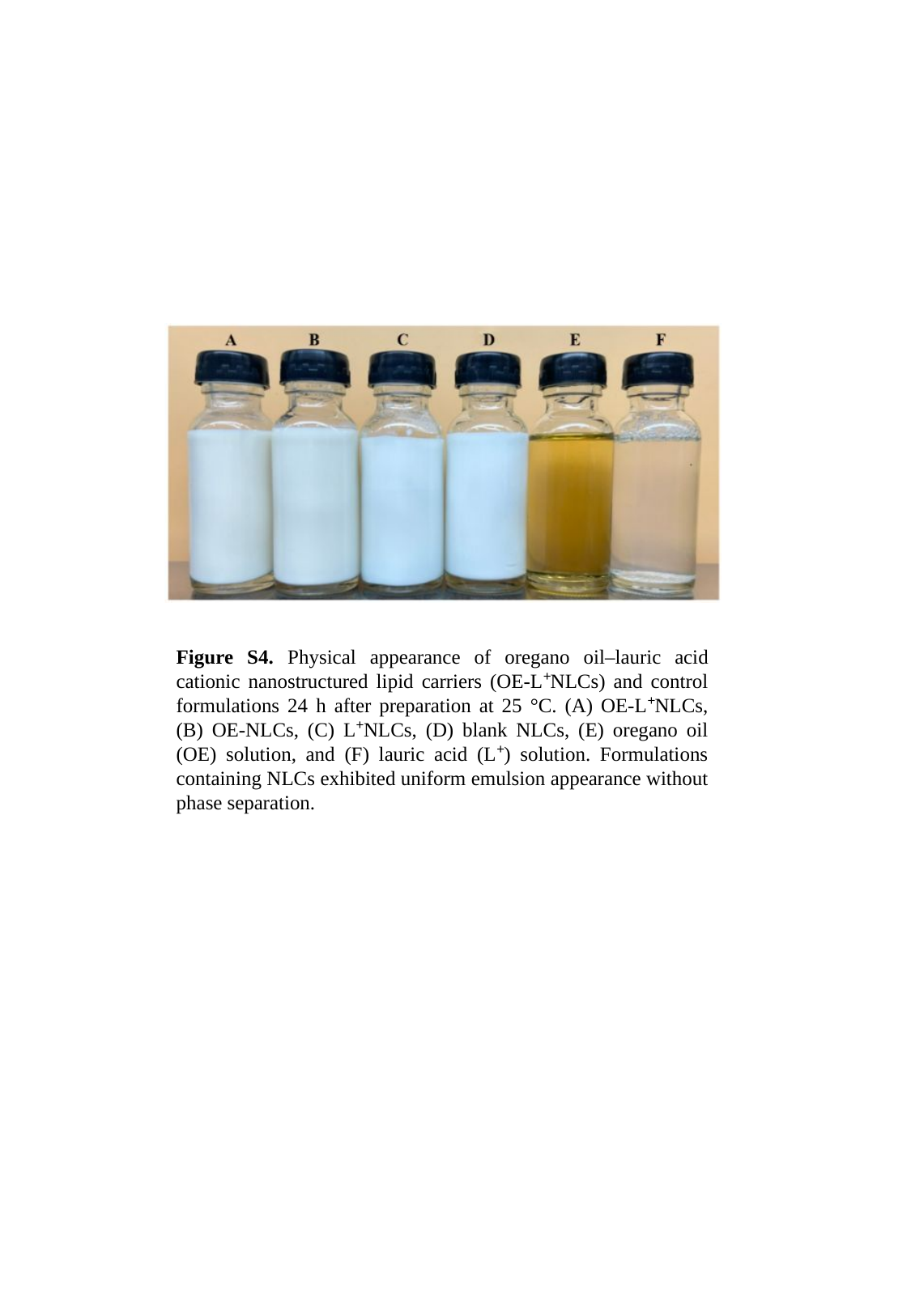

Figure S4. Physical appearance of oregano oil–lauric acid cationic nanostructured lipid carriers (OE-L⁺NLCs) and control formulations 24 h after preparation at 25 °C. (A) OE-L⁺NLCs, (B) OE-NLCs, (C) L⁺NLCs, (D) blank NLCs, (E) oregano oil (OE) solution, and (F) lauric acid (L⁺) solution. Formulations containing NLCs exhibited uniform emulsion appearance without phase separation.

## Slide 5
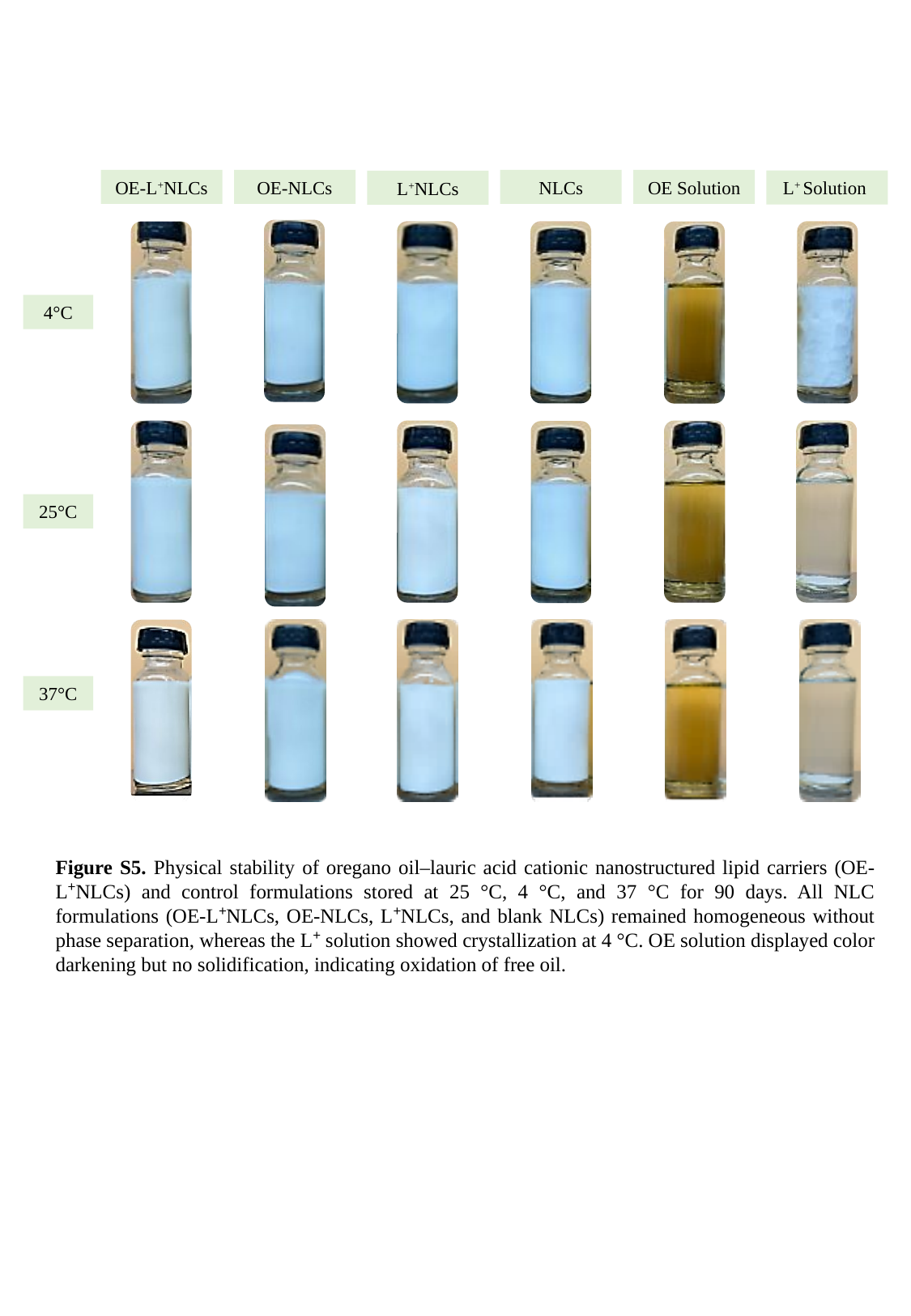

OE-L+NLCs
OE-NLCs
NLCs
OE Solution
L+ Solution
L+NLCs
4°C
25°C
37°C
Figure S5. Physical stability of oregano oil–lauric acid cationic nanostructured lipid carriers (OE-L⁺NLCs) and control formulations stored at 25 °C, 4 °C, and 37 °C for 90 days. All NLC formulations (OE-L⁺NLCs, OE-NLCs, L⁺NLCs, and blank NLCs) remained homogeneous without phase separation, whereas the L⁺ solution showed crystallization at 4 °C. OE solution displayed color darkening but no solidification, indicating oxidation of free oil.

## Slide 6
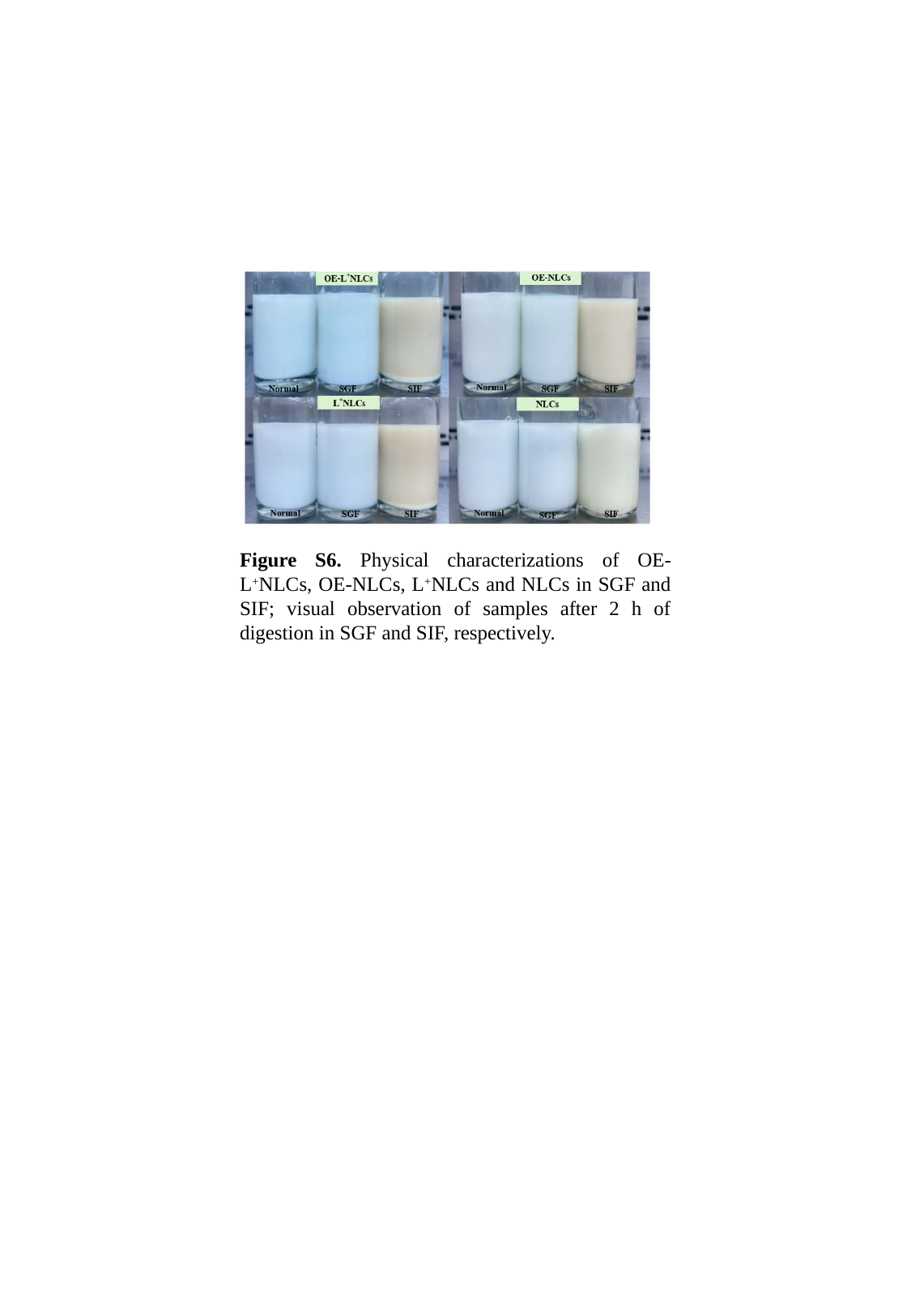

Figure S6. Physical characterizations of OE-L+NLCs, OE-NLCs, L+NLCs and NLCs in SGF and SIF; visual observation of samples after 2 h of digestion in SGF and SIF, respectively.
