# Supplementary material for: Antibacterial Activity and Safety of Oregano Oil–Lauric Acid Cationic Nanostructured Lipid Carriers in Nile Tilapia (Oreochromis niloticus)
Source: Animals (Basel). 2026 May 27;16(11):1639. doi: 10.3390/ani16111639 (PMC13255757; doi:10.3390/ani16111639)
Supplement: Supplementary file 1 [file animals-16-01639-s001.zip › Supplementary Table_OEL+NLC_Animals.pdf]

## Supplementary Table

**Table S1.** Detailed formulation composition of OE-$L^+$NLCs, OE-NLCs, $L^+$NLCs, and blank NLCs.

| **Formulations** | **Formulation in 100 mL (%w/w)** | | | | | | | | |
|---|---|---|---|---|---|---|---|---|---|
| | **$L^+$** | **OE** | **MCT** | **Span 20** | **Montanov 82** | **Tween 80** | **Glycerol monohydrate** | **Polyethylene glycol** | **$dH_2O$ or DMSO** |
| OE-$L^+$NLCs | 2 | 10 | 10-15 | 1-3 | 1-3 | 1-3 | 1-3 | 1-3 | Fill up to 100 mL |
| OE-NLCs | - | 10 | 10-15 | 1-3 | 1-3 | 1-3 | 1-3 | 1-3 | |
| $L^+$NLCs | 2 | - | 10-15 | 1-3 | 1-3 | 1-3 | 1-3 | 1-3 | |
| NLCs | - | - | 10-15 | 1-3 | 1-3 | 1-3 | 1-3 | 1-3 | |
| OE solution | - | 10 | - | - | - | - | - | - | |
| $L^+$ solution | 2 | - | - | - | - | - | - | - | |
